# Influence of Obesity on Peri-Implant Health: A Cross-Sectional Clinical and Biochemical Study

**DOI:** 10.3390/diagnostics16070965

**Published:** 2026-03-24

**Authors:** Mine Keskin, Meltem Zihni Korkmaz, Semih Alperen Bostan, Mehtap Atak

**Affiliations:** 1 Department of Periodontology, Faculty of Dentistry, Recep Tayyip Erdoğan University, 53100 Rize, Turkeysemihalperen.bostan@erdogan.edu.tr (S.A.B.); 2 Department of Medical Biochemistry, Faculty of Medicine, Recep Tayyip Erdoğan University, 53100 Rize, Turkey; mehtap.atak@erdogan.edu.tr

**Keywords:** peri-implantitis, obesity, tumor necrosis factor-alpha, interleukin-1 beta, matrix metalloproteinase-8

## Abstract

**Background:** Obesity is associated with alterations in the immune response through increased systemic inflammation. This systemic inflammatory state may increase the risk of peri-implantitis, a condition characterized by infection and tissue destruction around dental implants. Therefore, this cross-sectional clinical study aimed to investigate the association between obesity and peri-implant health. **Methods:** In this observational clinical study, a total of 80 patients were evaluated, including a peri-implant healthy non-obese control group (CG) (*n* = 20), peri-implantitis non-obese group (PG) (*n* = 20), peri-implant healthy obese group (OG) (*n* = 20), and peri-implantitis obese group (POG) (*n* = 20). Peri-implant clinical measurements (plaque index [PI], gingival index [GI], bleeding on probing [BOP], and probing depth [PD]) were obtained from the participants. In addition, tumor necrosis factor-alpha (TNF-α), interleukin-1 beta (IL-1β), and matrix metalloproteinase-8 (MMP-8) levels were measured in peri-implant crevicular fluid (PICF) samples. **Results:** PI, GI, BOP, and PD levels were significantly higher in the POG and PG than in the other groups (*p* < 0.05). PICF volume was found to be higher in the POG and PG than in the control group (*p* < 0.05). TNF-α levels increased significantly in all groups compared with the control group, and IL-1β levels were highest in the POG (*p* < 0.05). **Conclusions:** The findings of this cross-sectional study suggest a potential association between increased proinflammatory cytokine levels and altered peri-implant inflammatory responses in patients with obesity. Trial registration: This study was registered on ClinicalTrials.gov (Identifier: NCT07183163) on 18 September 2025 (retrospectively).

## 1. Introduction

Peri-implant inflammatory diseases are characterized by progressive inflammation affecting the gums and alveolar bone around the implant [1]. These diseases develop as a result of the host defense response triggered by microbial colonization and biomechanical loads, similar to periodontal diseases. In the pathogenesis of peri-implantitis, the host response is shaped by the interaction of microbial load and environmental (local) and systemic factors [2]. In this process, inadequate regulation of the host response may allow inflammation to extend beyond the peri-implant soft tissues, with accompanying progressive alveolar bone resorption and possible implant loss [3]. Local risk factors include poor oral hygiene, tobacco use, cement residues, and incompatible implant restorations, and conditions such as diabetes mellitus (DM), cardiovascular diseases (CVD), osteoporosis, and obesity stand out among systemic risk factors [4].

Obesity is a complex, multifactorial metabolic disease characterized by an excessive increase in body fat percentage and is usually defined in individuals with a body mass index (BMI) ≥ 30 kg/m^2^, with anthropometric indicators such as waist circumference frequently used to better characterize central adiposity [5,6]. In obese individuals, proinflammatory cytokines (e.g., tumor necrosis factor-alpha (TNF-α), interleukin-6 (IL-6)) and adipokines (e.g., leptin, resistin) are associated with persistent local and systemic inflammation and with alterations in host response that may be associated with the aggravation of chronic inflammatory processes [7,8]. Obesity and obesity-related inflammation have been associated with several chronic inflammatory diseases, such as CVD, non-alcoholic fatty liver disease, rheumatoid arthritis, and periodontitis, and may be linked to greater disease severity [9,10].

Although the biologic mechanisms underlying the association between obesity and peri-implantitis remain incompletely understood, the connection between increased adiposity and periodontal tissue destruction is a prominent topic in the recent literature [11,12]. The overexpression of proinflammatory cytokines in the bloodstream, associated with increased adipose tissue, may be linked to changes in immune response and to greater susceptibility to inflammatory conditions such as periodontitis and peri-implantitis [12,13]. Studies show that interleukin-1 beta (IL-1β), IL-6, the peri-implant plaque index (PI), bleeding on probing (BOP), and probing depth (PD) levels are high in individuals with obese peri-implantitis [14]. Similarly, Vohra et al. [13] reported an increase in PI, BOP, and PD values around the implant in individuals with obesity. In addition, obesity has been associated with alterations in bone metabolism, possibly through changes in the balance between osteoblast and osteoclast activity [12]. Obesity-related immune changes in periodontal tissues may be associated with modifications in the pocket environment and host defense mechanisms, potentially influencing subgingival microbial colonization [15]. These immunologic and metabolic changes may help explain the reported association between obesity and peri-implant diseases [13,14,16,17,18]. Several human clinical studies have demonstrated elevated peri-implant inflammatory parameters and increased proinflammatory cytokine levels in obese individuals compared with non-obese controls [13,14,18]. Previous human studies have investigated the association between obesity and peri-implant inflammatory parameters. Clinical investigations have reported higher plaque index (PI), bleeding on probing (BOP), probing depth (PD), and elevated levels of proinflammatory cytokines in obese individuals compared with non-obese controls. These findings suggest that obesity may be associated with poorer peri-implant tissue health, possibly in relation to increased systemic inflammation. However, it remains unclear whether obesity exerts an additive inflammatory burden superimposed on peri-implantitis or whether both conditions share common inflammatory pathways that mutually enhance tissue destruction. Given the cross-sectional nature of the present study, causal inferences cannot be established, and further longitudinal studies are required to better clarify the nature of this interaction [14]. Therefore, the aim of the present cross-sectional study was to evaluate TNF-α, IL-1β, and MMP-8 levels in peri-implant crevicular fluid (PICF) from obese and non-obese individuals with and without peri-implantitis to better understand the interaction between obesity and peri-implant inflammation.

## 2. Materials and Methods

### 2.1. Ethical Approval

Ethical approval was obtained from the Recep Tayyip Erdoğan University Non-Invasive Clinical Research Ethics Committee for this study (Approval No: 2022/117). The research was carried out in accordance with the ethical principles set out in the Declaration of Helsinki (2013 revision). All participants were informed in detail about the purpose and procedures of the study, and written consent was obtained from each participant voluntarily.

The study protocol, predefined outcome measures, and statistical analysis plan were established prior to data analysis. The trial was registered at ClinicalTrials.gov (Identifier: NCT07183163); registration was completed retrospectively due to administrative reasons.

### 2.2. Sample Size Calculation

The sample size of this study was calculated using the G*Power statistics program (ver.3.1.9.7). Accordingly, in the one-way analysis of variance (ANOVA) experimental design, when power (test power) was taken as 0.80, the effect size as 0.4 (F test effect size value range), and with type-1 error (α) as 0.05, a total of 76 samples with a minimum of 19 in each group was determined. The effect size (f = 0.40) was determined according to Cohen’s recommendations for large effect sizes in ANOVA designs [19]. Taking this into account, to increase the study’s power, four groups (*n* = 20) were formed, totaling 80 participants.

### 2.3. Study Population

A total of 80 participants aged 18–65 years who presented to the Periodontology Clinic of Recep Tayyip Erdoğan University and had implant treatment (osteointegration completed and prosthetic treatment) at least 48 months ago were included in the study. Participant recruitment and data collection were conducted between January 2023 and January 2024. As this was a cross-sectional clinical study, follow-up was not required beyond the single measurement time point. These participants were allocated to the four groups as follows: non-obese, peri-implant healthy control group (CG, *n* = 20); non-obese, peri-implantitis group (PG, *n* = 20); obese, peri-implant healthy group (OG, *n* = 20); obese, peri-implantitis group (POG, *n* = 20). The inclusion criteria were based on the diagnostic criteria recommended by the 2017 Periodontal Disease Classification [20] and the 2024 Turkish Endocrinology and Metabolism Association Obesity Diagnosis and Treatment Guideline [6]. The inclusion criteria were defined according to the 2017 World Workshop on the Classification of Periodontal and Peri-Implant Diseases and Conditions and the 2024 Turkish Endocrinology and Metabolism Association Obesity Diagnosis and Treatment Guideline. Clinically, peri-implant health was characterized by the absence of inflammatory signs such as bleeding or suppuration on probing and by stable crestal bone levels beyond physiologic remodeling. In contrast, peri-implantitis was defined by the presence of bleeding and/or suppuration on probing, a probing depth ≥6 mm, and radiographic evidence of bone loss ≥3 mm apical to the most coronal portion of the intraosseous implant component. Obesity was determined using both body mass index (BMI) and waist circumference (WC) measurements. Individuals were considered obese when BMI was ≥30 kg/m^2^ together with a waist circumference >100 cm in men and >90 cm in women. The additional evaluation of waist circumference allowed assessment of abdominal fat distribution in addition to overall body mass. Participants who reported tobacco use (including smokeless tobacco), regular alcohol consumption, pregnancy or lactation, systemic diseases such as acquired immune deficiency syndrome/HIV infection, kidney disorders, cardiovascular disease, or diabetes mellitus, as well as those who had received periodontal or peri-implant treatment within the previous six months, were excluded from the study. To minimize potential confounding related to implant characteristics, implant-related variables were standardized across the study population. All evaluated implants were placed using the same implant system with similar surface characteristics and were restored following comparable prosthetic protocols. In addition, only implants that had been in functional loading for at least 48 months were included in the analysis. This approach was adopted to reduce the potential influence of implant design and loading duration on peri-implant inflammatory parameters.

### 2.4. Clinical Peri-Implant Parameters

In the study, peri-implant measurements were made by a calibrated and trained clinician with no knowledge of the participants’ medical histories (S.A.B.). The examiner who performed the measurements was blinded to group allocation. Peri-implant status was evaluated using PI [21], gingival index (GI) [21], BOP [22], and PD. Radiographic bone loss (≥3 mm apical to the most coronal part of the implant) was measured using standardized periapical radiographs obtained with the parallel technique. Intra-examiner calibration was performed prior to analysis, yielding an intraclass correlation coefficient (ICC) of 0.90 or higher. Millimeter-precision measurements were made using a manual graded probe (UNC-15, Hu-Friedy, Chicago, IL, USA) from six sites (mesiobuccal, mid-buccal, distobuccal, distolingual/palatal, mid-lingual/palatal, and mesiolingual/palatal) on each implant. Bleeding on probing (BOP) was recorded dichotomously (presence/absence) at six sites per implant. The BOP score was calculated as the number of bleeding sites divided by the total number of examined sites per implant and expressed as a percentage. To minimize potential clustering effects and ensure statistical independence of observations, only one implant per participant was included in the analysis. For standardization purposes, the implant placed in the maxillary first molar region was consistently selected for evaluation in each patient. Therefore, the unit of analysis was the participant.

### 2.5. Collection of PICF (Peri-Implant Crevicular Fluid) Samples

Prior to the collection of PICF samples, the study area was isolated with cotton rolls and dried with light airflow for 10 s. Sterile paper strips (Periopaper^®^, Proflow Inc., New York, NY, USA) were placed in the sulcus for 30 s until minimal resistance was felt. Samples with blood or saliva contamination were not included in the study. Volume measurements of the collected samples were performed using electrical impedance with a Periotron 8010 device (Harco Electronics, Winnipeg, MB, Canada), and the results were recorded in microliters (μL). Then, four paper strips from each patient were transferred to sterile Eppendorf tubes containing 250 μL of phosphate-buffered saline (PBS). The tubes were placed in an ultra-low-temperature freezer (Thermo Fisher Scientific, Waltham, MA, USA) and stored at −80 °C until biochemical analyses could be performed.

### 2.6. Biochemical Analysis

The PICF samples in Eppendorf tubes were removed from the freezer 24 h prior to testing and thawed slowly, first at −20 °C and then at +4 °C. Just before analysis, the samples and reagents were brought to room temperature (18–25 °C). IL-1β, TNF-α, and MMP-8 analyses were performed using enzyme-linked immunosorbent assay (ELISA) according to the manufacturer’s instructions (Elabscience, Houston, TX, USA). All samples were analyzed in duplicate, and the mean values were used for statistical analysis. Although the analytical range of the MMP-8 ELISA kit is provided in ng/mL by the manufacturer, all measured concentrations were converted to pg/mL and reported consistently throughout the manuscript.

### 2.7. Statistical Analysis

The normality of the data distribution was assessed using the Shapiro–Wilk test. Homogeneity of variances was evaluated using Levene’s test when parametric tests were applied. Variables that met normality and homogeneity assumptions were analyzed using one-way ANOVA, whereas variables that did not meet these assumptions were analyzed using the Kruskal–Wallis test. The post hoc Bonferroni test was used to identify the group or groups that accounted for the difference. Pearson’s Chi-square analysis was used to examine the relationships between categorical variables. To test the relationship between continuous variables, Pearson’s correlation analysis was used when data were normally distributed, and Spearman’s correlation analysis was used when they were not normally distributed. The analyses were performed using IBM SPSS Statistics 25 (SPSS Inc., Chicago, IL, USA). Samples with cytokine concentrations below the assay’s lower limit of detection were excluded from the respective analyses. This resulted in a reduced sample size for IL-1β in some groups.

## 3. Results

### 3.1. Demographic and Anthropometric Characteristics

The demographic and anthropometric data of the participants are shown in Table 1. There was no statistically significant difference between the study groups in terms of age and sex (*p* > 0.05). In BMI and WC measurements, POG and OG showed a significant increase compared with the other groups (*p* < 0.05) (Table 1).

### 3.2. Clinical Parameters

The distribution of peri-implant clinical parameters between groups is shown in Table 2. PI, GI, BOP, and PD scores were found to be significantly higher in the PG and POG compared with the CG and OG (*p* < 0.05) (Table 2).

### 3.3. Biochemical Findings

The distribution of PICF volume, TNF-α, IL-1β, and MMP-8 levels between the groups is shown in Table 3. Accordingly, TNF-α levels were significantly lower in the CG than in the other groups (*p* < 0.05). IL-1β levels were significantly higher in the POG than in the other groups (*p* < 0.05). The mean MMP-8 measurement was significantly higher in the PG than in the POG and OG (*p* < 0.05). Although PICF volume levels were found to be higher in the POG and PG than in the control group, there was also a significant increase in the POG in the OG (*p* < 0.05) (Table 3).

### 3.4. Correlation Findings

TNF-α levels were positively correlated with GI, BOP, PD, IL-1β, MMP-8, PICF volume, BMI, and WC (*p* < 0.05). IL-1β levels were positively correlated with GI, BOP, PD, TNF-α, PICF volume, and BMI (*p* < 0.05). MMP-8 levels were positively correlated with BOP and TNF-α (*p* < 0.05). A positive correlation was found between BMI and WC (*p* < 0.05) (Table 4).

## 4. Discussion

To the best of our knowledge, no other study in the literature has measured TNF-α, IL-1β, and MMP-8 levels in PICF to investigate the relationship between obesity and peri-implantitis. According to our findings, the association between obesity and peri-implant inflammatory conditions may be partly related to increased proinflammatory cytokine levels.

In our study, PI, GI, BOP, and PD levels were elevated in obese and non-obese patients with peri-implantitis, while the association of obesity with these parameters appeared limited. In many studies in the literature, peri-implant tissues were evaluated [4,13,16,17]. PI, BOP, PD, and marginal bone loss (MBL) values were found to be higher in participants with obesity compared with those who were not obese. By contrast, Alasgah et al. [18] reported that obesity did not significantly alter PD and MBL parameters in peri-implant tissues. In their study of patients with peri-implantitis, Elsadek et al. [14] found that obesity was associated with increased periodontal clinical parameters.

The current approach to diagnosing peri-implantitis relies on clinical and radiographic evaluations, but although these parameters are useful for identifying advanced disease, they may not be sufficiently sensitive for detecting early peri-implant inflammation or assessing patient sensitivity. At this point, it is thought that the biomarkers examined in PICF, which can be collected non-invasively, may complement the clinical parameters [23].

TNF-α is produced by immune cells, such as macrophages and T and B lymphocytes, in response to bacterial infection or tissue damage. It plays a role in bone loss by increasing osteoclastic activity in diseases such as periodontitis and rheumatoid arthritis, which are characterized by chronic inflammation [23,24]. Our findings are consistent with studies reporting increased TNF-α levels in PIOS samples obtained from patients with peri-implantitis [25,26,27]. However, obesity has had only a limited effect on the increase in TNF-α observed in peri-implantitis. In the literature, only a limited number of studies have examined the relationship between peri-implant disease and obesity on TNF-α levels. Obesity is characterized by a chronic low-grade systemic inflammatory state in which expanded adipose tissue secretes proinflammatory cytokines such as TNF-α. Accordingly, the elevated TNF-α levels observed in obese individuals without peri-implantitis in the present study may reflect the systemic inflammatory burden associated with obesity rather than local peri-implant pathology alone [28,29,30].

In an animal study by Coelho et al. [31] examining osteointegration and biochemical parameters, it was reported that obesity did not significantly increase TNF-α levels. Lumbikananda et al. [23] reported that an increase in IL-1β in PICF samples was accompanied by an increase in TNF-α. Supporting our findings, a statistically significant positive correlation was found between TNF-α and GI, BOP, PD, PICF volume, BMI, IL-1β, and WC measurements.

IL-1β, a proinflammatory cytokine produced mainly by monocytes and macrophages, is involved in the aggregation of immune cells to the site of infection. Many cytokines, especially IL-1β, are involved in directing tissue destruction in both periodontitis and periimplantitis [23,32,33]. Studies report increased IL-1β levels in PICF samples from patients with peri-implantitis [27,34,35]. IL-1β levels, which we found to be high in the obese peri-implantitis group, were consistent with those reported by Elsadek et al., who used a similar methodology [14]. Moreover, other studies examining the effects of obesity on peri-implant tissues reported increased IL-1β levels in PICF [16,18] and saliva [17] samples. Considering the positive correlation between IL-1β levels and the clinical parameters GI, BOP, and PD, these findings may suggest that obesity is associated with a more pronounced peri-implant inflammatory profile.

MMP-8 is one of the major collagenolytic enzymes involved in the destruction and progression of periodontal and peri-implant tissue [23]. MMP upregulation has been associated with irreversible peri-implant connective tissue destruction [36]. The level of MMP-8 in PICF and saliva is a potential biomarker for diagnosing peri-implantitis and assessing its inflammatory development [23,37]. Although there are many studies in the literature [38,39,40] reporting MMP-8 levels that increase with periimplantitis [38,39,40] or show no significant change [32], there is no study directly evaluating MMP-8 levels in patients with peri-implantitis, including the obesity factor. According to our findings, the highest MMP-8 level was measured in the group with peri-implantitis, but who were not obese. In the present study, MMP-8 levels were found to be higher in the non-obese peri-implantitis group, which may initially appear inconsistent with the expectation that obesity would further increase inflammatory parameters. However, MMP-8 is mainly released from activated neutrophils and reflects local collagen degradation in peri-implant tissues [41]. In contrast, obesity is primarily associated with chronic low-grade systemic inflammation mediated by cytokines [42,43]. Therefore, MMP-8 levels may reflect local peri-implant disease activity rather than systemic inflammatory status [40]. Importantly, large population-based data demonstrate an inverse association between obesity and circulating MMP-8 levels. Therefore, obesity-related chronic inflammation may not necessarily be accompanied by increased neutrophil-driven MMP-8 expression. Although statistically significant, the absolute differences observed were relatively small and should be interpreted cautiously in terms of clinical relevance [44]. This was the case for Youn Ho Shin et al. [45]. TNF-α levels were elevated in peri-implantitis groups irrespective of obesity status, suggesting that this cytokine primarily reflects disease-associated inflammation rather than an obesity-specific effect. In contrast, IL-1β levels were highest in the obese peri-implantitis group, which may suggest an association between obesity and enhanced peri-implant inflammatory activity.

An unexpected finding of the present study was that MMP-8 levels were highest in the non-obese peri-implantitis group. Clinical peri-implant parameters—including plaque index (PI), gingival index (GI), bleeding on probing (BOP), probing depth (PD), and PICF volume—were comparable between peri-implantitis groups. In addition, implant-related variables such as implant type and functional duration were similar across groups. Therefore, the observed difference in MMP-8 levels is unlikely to be fully explained by measured clinical inflammatory parameters. This finding partially challenges the assumption that obesity uniformly amplifies all peri-implant inflammatory mediators, suggesting a more complex interaction between systemic metabolic status and local tissue-destructive pathways.

As MMP-8 reflects collagen degradation and neutrophil activity at the molecular level, the lack of parallel changes with clinical inflammatory indices or systemic cytokine levels observed in the present study suggests that these pathways may not be uniformly regulated. While statistically significant, the magnitude and clinical implications of the observed differences should be interpreted with caution and require further evaluation in longitudinal studies. These findings indicate that the relationship between systemic inflammatory status and local peri-implant tissue responses may not be directly parallel and warrant further investigation. In addition, potential confounding variables such as dietary habits, frequency of professional maintenance, and individual oral hygiene practices were not quantitatively assessed. These factors may influence cytokine expression and peri-implant inflammatory responses. Therefore, the findings of this cross-sectional study should be interpreted with caution. Implant-related variables such as implant brand, surface characteristics, and duration of function were standardized across groups as described in the Section 2; however, their potential influence on the observed inflammatory profiles cannot be completely excluded [41,46].

Among our limitations are the lack of obesity classification in the study group design, the small sample size, and the limited number of PICF samples collected, which limited our ability to examine more parameters. Obesity subclasses were not analyzed separately, and central adiposity was not evaluated independently from BMI. In addition, pooling four PICF strips obtained from the same implant provided an overall inflammatory profile of the implant site but did not allow for surface-specific analysis. Several limitations of this study should be acknowledged. The cross-sectional design precludes causal inferences. The trial registration was completed retrospectively; however, the study protocol and predefined outcome measures were established prior to data analysis. Obesity subclasses were not analyzed separately. Although both BMI and waist circumference were recorded, participants were classified as obese only when both criteria were met; therefore, the independent effect of central obesity was not separately evaluated. The biochemical assessment was limited to TNF-α, IL-1β, and MMP-8, which may not fully reflect the multifactorial inflammatory processes underlying peri-implant tissue pathology. Finally, multivariable adjustment was not performed to control for potential confounding variables. Future longitudinal studies incorporating broader biomarker profiling and multivariate analytical approaches are warranted.

## 5. Conclusions

The present observational cross-sectional study suggests that obesity may be associated with altered peri-implant inflammatory responses and higher levels of certain proinflammatory mediators. However, due to the cross-sectional design, no causal inferences can be drawn. Further studies with larger sample sizes evaluating the microbiologic and immune-inflammatory aspects of peri-implant regions in patients with obesity are warranted to clarify these associations.

## Figures and Tables

**Table 1:** Evaluation of demographic anthropometric parameters between the groups.

	Groups	*n*	Median (Min–Max)	Mean ± SD	*p*
Age	Control	20	56 (37.0–64.0)	53.450 ± 7.8369	0.244
	PG	20	51 (29.0–64.00)	50.000 ± 8.4914	
	OG	20	55.5 (47.0–62.00)	54.750 ± 5.2302	
	POG	20	54.5 (47.0–63.00)	54.600 ± 4.8817	
BMI (kg/m^2^)	Control ^a^	20	25.4 (19.6–29.72)	25.270 ± 2.9895	<0.001 *
	PG ^a^	20	25.415 (21.5–29.30)	25.961 ± 2.3758	
	OG ^b^	20	31.3 (30.0–37.50)	31.977 ± 2.0077	
	POG ^b^	20	33.08 (29.2–41.34)	33.500 ± 3.7264	
WC (cm)	Control ^a^	20	89 (34.0–99.00)	87.150 ± 14.755	<0.001 *
	PG ^a^	20	92 (68.0–100.0)	88.950 ± 10.112	
	OG ^b^	20	102 (92.0–120.0)	103.05 ± 8.1011	
	POG ^b^	20	106 (94.0–133.0)	109.05 ± 12.634	
Gender (F/M) (*n*)	Control	7/13			0.810
	PG	9/11			
	OG	9/11			
	POG	10/10			

PG: Peri-implantitis and non-obese group, OG: obese and peri-implant healthy group, POG: obese and peri-implantitis group, *n*: number of participants in groups, Min–Max: minimum–maximum, BMI: body mass index (kg/m^2^), WC: waist circumference (cm), cm: centimeter, kg: kilogram, m: meter, F: female, M: male, SD: Standard deviation. ^a,b^: different letters indicate significant difference between groups, *: *p* < 0.05.

**Table 2:** Evaluation of clinical parameters between the groups.

	Groups	*n*	Median (Min–Max)	Mean ± SD	*p*
PI (mm)	Control ^a^	20	0.9250 (0.30–1.99)	1.0080 ± 0.4200	<0.001 *
	PG ^b^	20	2.4850 (1.58–2.85)	2.3470 ± 0.3850	
	OG ^a^	20	1.2550 (0.58–2.84)	1.5690 ± 0.7744	
	POG ^b^	20	2.4050 (1.53–2.78)	2.3905 ± 0.3144	
GI	Control ^a^	20	0.6050 (0.22–1.86)	0.6745 ± 0.3424	<0.001 *
	PG ^b^	20	2.5950 (1.83–2.95)	2.5010 ± 0.3433	
	OG ^a^	20	0.5650 (0.23–0.88)	0.5400 ± 0.1711	
	POG ^b^	20	2.5900 (1.70–2.95)	2.5935 ± 0.2687	
BOP	Control ^a^	20	2.6000 (1.30–8.70)	3.0230 ± 1.52162	<0.001 *
	PG ^b^	20	91.5000 (41.3–96.6)	86.795 ± 12.723	
	OG ^a^	20	3.0500 (1.90–5.10)	3.3350 ± 0.8591	
	POG ^b^	20	84.5000 (30.4–96.8)	82.215 ± 15.557	
PD (mm)	Control ^a^	20	2.0500 (1.13–2.98)	1.9425 ± 0.5456	<0.001 *
	PG ^b^	20	3.3300 (2.45–5.02)	3.4190 ± 0.5574	
	OG ^a^	20	2.4300 (1.97–2.92)	2.4055 ± 0.2918	
	POG ^b^	20	3.5750 (3.01–5.20)	3.7620 ± 0.6820	

PG: Peri-implantitis and non-obese group, OG: obese and peri-implant healthy group, POG: obese and peri-implantitis group, *n*: number of participants in groups, Min–Max: minimum–maximum, PI: plaque index, GI: gingival index, BOP: bleeding on probing, PD: probing depth (mm), SD: standard deviation, mm: millimeter. ^a,b^: Different letters indicate significant difference between groups, *: *p* < 0.05.

**Table 3:** Evaluation of biochemical parameters between groups.

	Groups	*n*	Median (Min–Max)	Mean ± SD	*p*
TNF-α (pg/mL)	Control ^a^	20	9.7500 (5.29–24.86)	11.8607 ± 5.99657	<0.001 *
	PG ^b^	20	19.2857 (9.29–26.07)	18.3393 ± 5.03675	
	OG ^b^	20	18.3571 (8.64–23.00)	17.8250 ± 3.73674	
	POG ^b^	20	19.8571 (8.50–26.14)	19.7679 ± 4.62898	
IL-1β (pg/mL)	Control ^a^	11	5.3425 (0.33–9.23)	4.2540 ± 3.27867	<0.001 *
	PG ^a^	19	6.0000 (0.16–28.16)	9.5717 ± 8.76190	
	OG ^a^	18	5.2466 (0.85–30.14)	7.0776 ± 6.79085	
	POG ^b^	18	25.3562 (4.82–61.45)	25.9954 ± 15.3724	
MMP-8 (pg/mL)	Control ^a,b^	20	270.4963 (138.3–275.8)	257.718 ± 34.3240	0.006 *
	PG ^a^	20	273.0148 (266.9–276.8)	272.555 ± 3.09399	
	OG ^b^	20	269.2000 (261.7–274.5)	268.174 ± 3.81011	
	POG ^b^	20	268.2370 (254.2–275.2)	268.618 ± 5.01683	
PICF volume (μL)	Control ^a^	20	0.3600 (0.12–0.86)	0.3935 ± 0.21122	<0.001 *
	PG ^b,c^	20	0.6900 (0.25–1.49)	0.8095 ± 0.41500	
	OG ^a,c^	20	0.4900 (0.17–1.49)	0.6075 ± 0.39037	
	POG ^b^	20	0.9250 (0.53–1.65)	0.9585 ± 0.32328	

PG: Peri-implantitis and non-obese group, OG: obese and peri-implant healthy group, POG: obese and peri-implantitis group, *n*: number of participants in groups, Min–Max: minimum–maximum, TNF-α: tumor necrosis factor alpha, IL-1β: interleukin 1 beta, MMP-8: matrix metalloproteinase-8, PICF: peri-implant crevicular fluid. ^a,b,c^: Different letters indicate significant difference between groups, pg/mL: picograms per milliliter, μL: microliter, *: *p* < 0.05.

**Table 4:** Pearson correlation coefficients among clinical, biochemical, and anthropometric parameters.

	**PI**	**GI**	**BOP**	**PD**	**TNF-α**	**IL-1β**	**MMP-8**	**PICF**	**Age**	**BMI**	**WC**
PI		0.647 (<0.001)	0.595 (<0.001)	0.566 (<0.001)	0.196 (0.081)	0.205 (0.098)	0.049 (0.666)	0.341 (0.002)	−0.156 (0.166)	0.149 (0.188)	0.158 (0.161)
GI			0.765 (<0.001)	0.745 (<0.001)	0.288 (0.010)	0.412 (0.001)	0.105 (0.354)	0.511 (<0.001)	−0.056 (0.622)	0.075 (0.511)	0.106 (0.351)
BOP				0.783 (<0.001)	0.311 (0.005)	0.352 (0.004)	0.228 (0.042)	0.495 (<0.001)	−0.074 (0.512)	0.132 (0.243)	0.052 (0.646)
PD					0.357 (0.001)	0.484 (<0.001)	0.192 (0.088)	0.443 (<0.001)	−0.028 (0.806)	0.278 (0.013)	0.218 (0.052)
TNF-α						0.525 (<0.001)	0.289 (0.009)	0.355 (0.001)	0.030 (0.790)	0.257 (0.021)	0.222 (0.048)
IL-1β							0.042 (0.737)	0.380 (0.002)	0.073 (0.558)	0.404 (0.001)	0.165 (0.186)
MMP-8								−0.024 (0.832)	−0.130 (0.251)	−0.245 (0.028)	−0.210 (0.062)
PICF									−0.159 (0.159)	0.271 (0.015)	0.140 (0.217)
Age										0.149 (0.188)	0.082 (0.471)
BMI											0.698 (<0.001)
WC											

PI: Plaque index, GI: gingival index, BOP: bleeding on probing, PD: probing depth (mm), TNF-α: tumor necrosis factor alpha, IL-1β: interleukin 1 beta, MMP-8: matrix metalloproteinase-8, PICF: peri-implant crevicular fluid, BMI: body mass index (kg/m^2^), WC: waist circumference (cm), cm: centimeter, kg: kilogram, m: meter.

## Data Availability

The data presented in this study are available on request from the corresponding author. The data are not publicly available due to ethical and privacy restrictions.

## Abbreviations

The following abbreviations are used in this manuscript:

BMI	Body mass index
BOPJ	Bleeding on probing
CVD	Cardiovascular diseases
DM	Diabetes mellitus
ELISA	Enzyme-linked immunosorbent assay
GI	Gingival index
IL-1β	Interleukin-1 beta
IL-6	Interleukin-6
PBS	Phosphate-buffered saline
PI	Plaque index
PPD	Probe pocket depth
PICF	Peri-implant crevicular fluid
MMP-8	Matrix metalloproteinase-8
TNF-α	Tumor necrosis factor-alpha
WC	Waist circumference
